# Supplementary material for: Impact of the COVID-19 Pandemic on Tumor Stage and Pathohistological Parameters of Vulvar Cancer
Source: J Clin Med. 2024 Jul 11;13(14):4058. doi: 10.3390/jcm13144058 (PMC11277637; doi:10.3390/jcm13144058)
Supplement: Supplementary file 1 [file jcm-13-04058-s001.zip › jcm-3056156-supplementary.pdf]

Title: Impact of the COVID-19 Pandemic on Tumor Stage and Pathohistological Parameters of Vulvar Cancer

Supplementary Material

**inclusion criteria**	**exclusion criteria**
squamous cell carcinoma of the vulva	high grade dysplasia (HSIL, dVIN)
correctly classified TNM staging (p/cTNM)	bioptical diagnosis only
vulvar surgery	insufficient pathological diagnostics (either due to tissue-related / artifact-related affections)
	tumor entities of the vulva other than squamous cell carcinoma
	tumor entities of the vagina or anus
	recurrence tumors
	patients with solely palliative-reductive surgery

Supplementary Table S1. List of a priori defined inclusion and exclusion criteria.

**tumor data within the pre-COVID cohort; number of sample (n = 30)**	max. tumor diameter (cm)	keratinization	minimal distance to resection margin (cm)
1	3,2	keratinizing	R1
2	1,7	non keratinizing	0,2
3	6,5	keratinizing	R1
4	5,4	keratinizing	0,1
5	6,3	keratinizing	R1
6	3,3	non keratinizing and low differentiation	0
7	4,2	non keratinizing and low differentiation	0,1
8	1,2	non keratinizing	0,3
9	1,7	keratinizing	0,2
10	6,9	keratinizing	0,5
11	1,5	keratinizing	0,4

12	3,5	non keratinizing and low differentiation	0,2
13	3,9	keratinizing	0,4
14	1,5	non keratinizing	0,5
15	2	keratinizing	0,3
16	5,2	keratinizing	R1
17	4,1	keratinizing	0,5
18	2,5	non keratinizing and low differentiation	0
19	0,55	non keratinizing	0,5
20	6,5	keratinizing	0,5
21	1,5	keratinizing	0,2
22	8	non keratinizing	R1
23	4	keratinizing	0,5
24	1,1	keratinizing	0,1
25	3,8	keratinizing	0,1
26	0,9	keratinizing	R1
27	8	keratinizing	0,1
28	1,5	keratinizing	0,5
29	0,5	not defined	0,5
30	3,8	not defined	0,2

Supplementary Table S2. Information about histomorphological tumor aspects within the pre- COVID cohort.

**tumor data within the COVID cohort; number of sample (n = 23)**	max. tumor diameter (cm)	keratinization	minimal distance to resection margin (cm)
1	3	non keratinizing	0,2
2	3	non keratinizing	0,1
3	1,6	not defined	0,3
4	2	non keratinizing	<0,1
5	not defined	non keratinizing	0,7
6	3	not defined	<0,1
7	2,6	not defined	0,3

8	2,9	not defined	0,4
9	2,6	not defined	0,3
10	3,7	not defined	0,5
11	not defined	non keratinizing	0,1
12	1,1	not defined	<0,1
13	1,4	not defined	0,5
14	8,2	non keratinizing	R1
15	0,7	not defined	0,2
16	1,8	not defined	0,3
17	0,6	non keratinizing	R1
18	0,5	non keratinizing	R1
19	1,8	non keratinizing	0,15
20	0,6	non keratinizing	0,2
21	0,9	non keratinizing	R1
22	1,7	non keratinizing	0,3
23	3	not defined	0,6

Supplementary Table S3. Information about histomorphological tumor aspects within the COVID cohort.

**tumor data within the post-COVID cohort; number of sample (n = 37)**	max. tumor diameter (cm)	keratinization	minimal distance to resection margin (cm)
1	0,6	keratinizing	0,8
2	11,6	keratinizing	0,2
3	2,5	not defined	R1
4	1,6	keratinizing	0,4
5	not defined	keratinizing	not defined (R1)
6	2,3	keratinizing	0,5
7	4,2	keratinizing	R1
8	7,8	keratinizing	0,1
9	not defined	not defined	0,4
10	0,9	not defined	0,5
11	not defined	keratinizing	0,1
12	1,2	keratinizing	0,1
13	0,7	keratinizing	0,5

14	0,9	keratinizing	not defined (R1)
15	4,5	keratinizing	0,1
16	4,8	keratinizing	0,5
17	not defined	keratinizing	R1
18	0,7	not defined	R1
19	1,2	keratinizing	0,6
20	1	not defined	0,1
21	0,2	not defined	0,2
22	2,1	not defined	0,1
23	0,9	keratinizing	0,2
24	2,3	keratinizing	0,5
25	4,5	not defined	R1
26	2,4	keratinizing	<0,1
27	not defined	not defined	0,1
28	2,1	keratinizing	<0,5
29	9,6	non keratinizing	0,1
30	2,9	keratinizing	0,2
31	not defined	keratinizing	0,4
32	4,1	keratinizing	R1
33	not defined	not defined	0,5
34	0,5	not defined	0,2
35	2,2	not defined	0,015
36	1,8	keratinizing	0,5
37	3,2	not defined	0,1

Supplementary Table S4. Information about histomorphological tumor aspects within the post-COVID cohort.
